# Use of a Cannulated, Percutaneous Expandable Reamer for Physeal Sparing Excision of a Femoral Head Chondroblastoma

**DOI:** 10.5435/JAAOSGlobal-D-23-00012

**Published:** 2023-09-12

**Authors:** Mallory P. Gersh, Benjamin Z. Mendelson, Anthony Judice, Albert J. Aboulafia

**Affiliations:** From the Wake Forest University, Winston-Salem, NC (Ms. Gersh); the West Virginia University School of Medicine, Morgantown, WV (Mr. Mendelson); the Vanderbilt University Medical Center, Nashville, TN (Dr. Judice); the Georgetown University School of Medicine, Washington, DC (Dr. Aboulafia); and the MedStar Georgetown/MedStar Franklin Square, and Sinai Hospital, University of Maryland School of Medicine, Baltimore, MD (Dr. Aboulafia).

## Abstract

The treatment of chondroblastoma in the epiphysis of the femoral head in skeletally immature individuals is challenging and often requires surgical hip dislocation. We present a unique method of percutaneous use of an expandable reamer (X-REAM, Wright Medical) to treat a chondroblastoma of the femoral head in a 9-year-old boy without requiring surgical hip dislocation. The described technique provides access to the tumor in the proximal femoral epiphysis and local tumor control. However, the approach involves placing a cannula through the epiphyseal plate, resulting in partial premature epiphyseal closure. At 5 years after surgery, the patient has an asymptomatic leg-length discrepancy and radiographic evidence of premature physeal closure, but no restrictions on activity or evidence of local recurrence.

A percutaneous expandable reamer can be used to treat chondroblastoma of the femoral head while avoiding surgical hip dislocation.

Chondroblastoma is a rare, benign tumor typically found in the epiphyses and apophyses of long bones.^1,2^ Chondroblastomas represent 1% to 2% of primary biopsy-analyzed bone tumors.^1,3^ Although chondroblastomas are benign, they can “metastasize” to the lung in a condition known as “benign metastasizing chondroblastoma.” Rarely, chondroblastoma can undergo malignant transformation and metastasis, which can be fatal.^1,2^ The most common symptoms of chondroblastoma are pain surrounding the affected joint with accompanying loss of motion.^1,4^ Treatment of chondroblastoma is often surgical given the symptoms and its locally aggressive nature. Curettage and bone grafting of the remaining cavity are the mainstays of orthopaedic surgical treatment.^3,5^ While percutaneous ablation techniques have been used to treat chondroblastoma, complications include subchondral bone infarction, chondrolysis, and recurrence.^6^ Unfortunately, these lesions often occur in inconvenient, tenuous regions where care is required to avoid damaging the surrounding physis and articular surface. Well-founded concern for the open physis often leads to inadequate curettage/resection of the tumor bed, which portends a higher rate of local recurrence.^1,4,5^ To limit damage to the open capital femoral physis while performing extensive intralesional curettage, we used a percutaneous, cannulated, expandable reamer. This technique limits damage to the open physis while enabling extensive curettage of the defect. It also obviates the need for surgical hip dislocation to gain access to the capital femoral epiphysis. The technique provides access to the tumor in the proximal femoral epiphysis and local tumor control. However, the approach involves placing a cannula through the epiphyseal plate, resulting in partial premature epiphyseal closure.

The patient and his guardian provided consent for publication of this case. The authors have no affiliation with the company that makes the reamer and have received nothing of value from the manufacturer.

## Case Report

A 9-year-old boy presented to an orthopaedic oncology clinic with right hip pain and a progressive limp for 3 months. He was an avid hockey player, but pain prevented him from competing. Nonsteroidal anti-inflammatory medications and rest improved the pain, but not to a satisfactory level. On physical examination, the right hip was moderately irritable with gentle internal and external rotation. The right hip had 170° of external rotation and 80° of internal rotation limited by pain; the left hip had 175° of external rotation and 95° of internal rotation. The patient had some laxity of both wrists and knees. He had an antalgic gait with decreased stance time on the right side.

**Figure 1:**
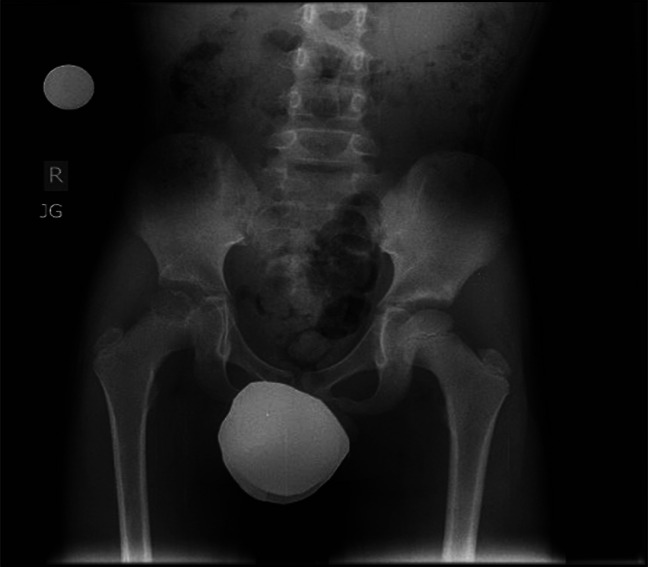
AP radiograph showing a well-defined radiolucent lesion in the epiphysis of the right proximal femur.

**Figure 2:**
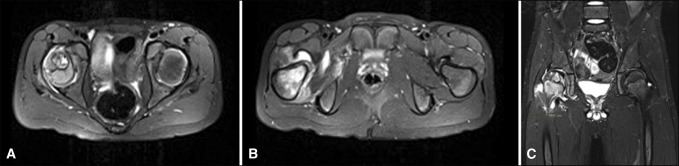
**A,** Radiograph showing axial T2 (repetition time 3230ms, echo time 4800ms) MRI of the pelvis revealing a well-defined lesion in the femoral epiphysis with a cystic component that measures 19.3 × 17.7 mm. **B,** Radiograph shows hyperintense marrow changes diffusely within the femoral head with associated joint effusion and soft-tissue edema. **C,** Radiograph showing coronal inversion recovery MRI (repetition time 4650ms, echo time 4600ms) demonstrating a well-defined lesion with1 hypointense lesion in the epiphysis with a sclerotic border and extensive marrow bright signal within the metaphysis and joint effusion. MRI = magnetic resonance imaging.

Radiographs showed a 2.3 × 1.7 cm lytic lesion with irregular margins in the epiphysis of the right femoral head (Figure 1). Magnetic resonance imaging of the pelvis and right hip showed a 2.0 × 2.0 cm cystic lesion in the right femoral epiphysis with associated joint effusion, extensive marrow edema, and soft-tissue edema (Figure 2). An obvious chondroid component was not identified. A CT scan showed a 1.3 × 2.8 cm lytic lesion in the anterolateral margin of the right femoral epiphysis with irregular borders. Whole-body technetium bone scan and chest radiograph were negative. Given these findings, a presumptive diagnosis of chondroblastoma was made with a differential diagnosis including infection, chondromyxoid fibroma, and giant cell tumor of bone. Biopsy and frozen section were recommended with the understanding that, if chondroblastoma was diagnosed at the time of biopsy, treatment would proceed.

**Figure 3:**
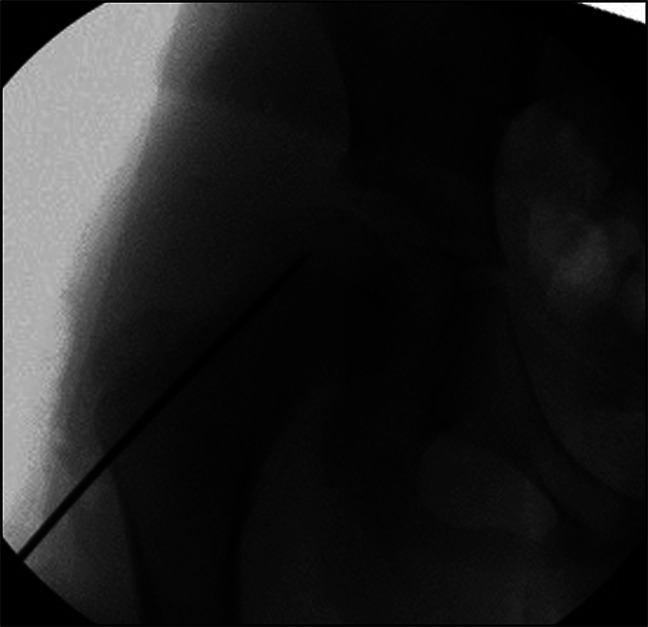
Intraoperative fluoroscopic image demonstrating the guide pin position into the epiphyseal lesion.

**Figure 4:**
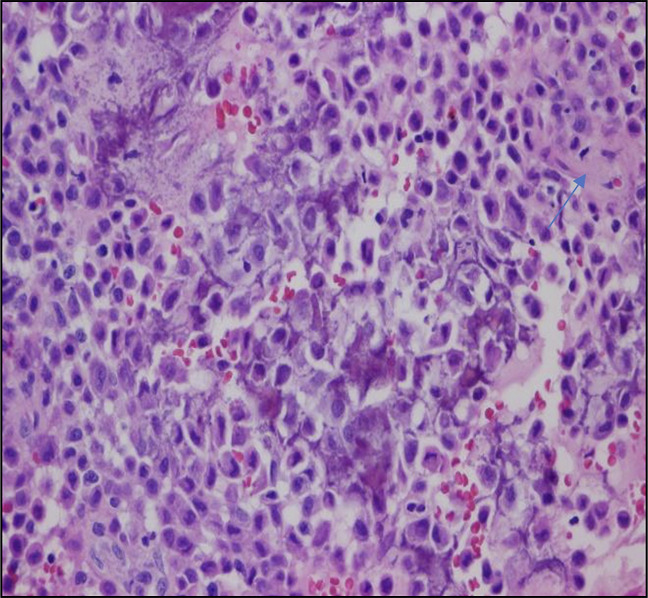
Image showing a microscopic specimen (hematoxylin and eosin stain) at ×100 magnification. The tumor is composed of round and polyhedral chondroblasts with abundant eosinophilic cytoplast and well-defined borders. The chondroid matrix is abundant (arrow).

**Figure 5:**
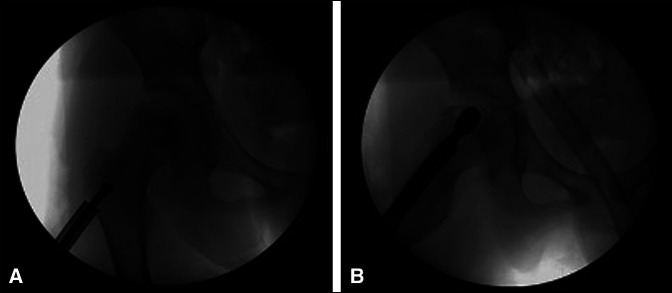
**A,** Radiograph showing a percutaneous expandable reamer system (X-REAM, Wright Medical) inserted through the cannula in a “closed position.” **B,** Radiograph showing the reamer within the lesion in the “open” position.

The patient was placed supine on a radiolucent table. An incision was made, extending from the prominence of the greater trochanter distally for 5 cm. The dissection was carried down through the subcutaneous tissue, and the deep fascia was incised in line with the incision. The vastus lateralis was then elevated anteriorly, and under fluoroscopic guidance, a guide pin was inserted laterally, up the femoral neck, across the physis, and into the epiphyseal lesion (Figure 3). Intraoperative fluoroscopy was used to confirm the position of the 3.2-mm guide pin in orthogonal planes. The tissue protector was then placed over the guidewire. Next, the 9-mm cannulated drill bit was placed over the guide pin and advanced to within 5 mm of the subchondral surface and the center of the tumor. A Lukens trap was attached to a Frazier suction tip, and the collected specimens were taken for frozen-section pathology analysis. A diagnosis of chondroblastoma was made (Figure 4).The drill bit and guidewire were removed, and the working cannula and obturator were then placed through the soft-tissue protector well into the femoral neck. The obturator and soft-tissue protector were removed, leaving the working cannula in place. The percutaneous expandable reamer was then passed through the working cannula, and the position was confirmed using fluoroscopy. The blade control knob was used to expand the blades (Figure 5). Only one size of blade is available with a maximum expansion of 2.1 cm.

**Figure 6:**
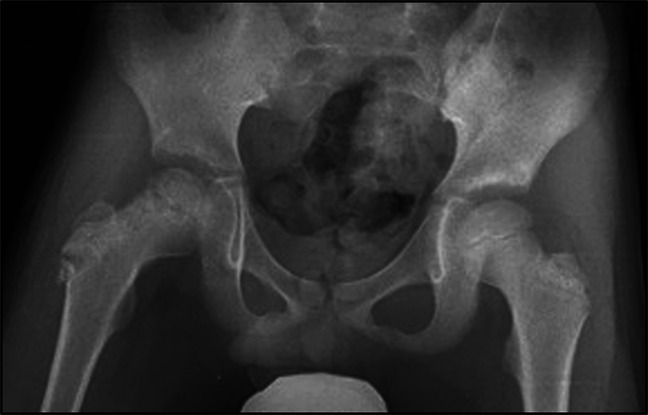
AP radiograph of the pelvis obtained 2 weeks after surgery showing postoperative changes and bone graft material filling the lesion within the epiphysis and the surgical tract within the femoral neck.

A series of curved curettes were then inserted through the channel to perform curettage of the remaining cavity. The cannula was then reinserted in the channel, and the cavity was backfilled with allograft cancellous chips and demineralized bone matrix. Intermittent continuous fluoroscopic evaluation demonstrated maintenance of the sphericity of the femoral head without extravasation of the graft into the hip joint (Figure 6). The wound was closed in layers, and a sterile dressing was applied.

**Figure 7:**
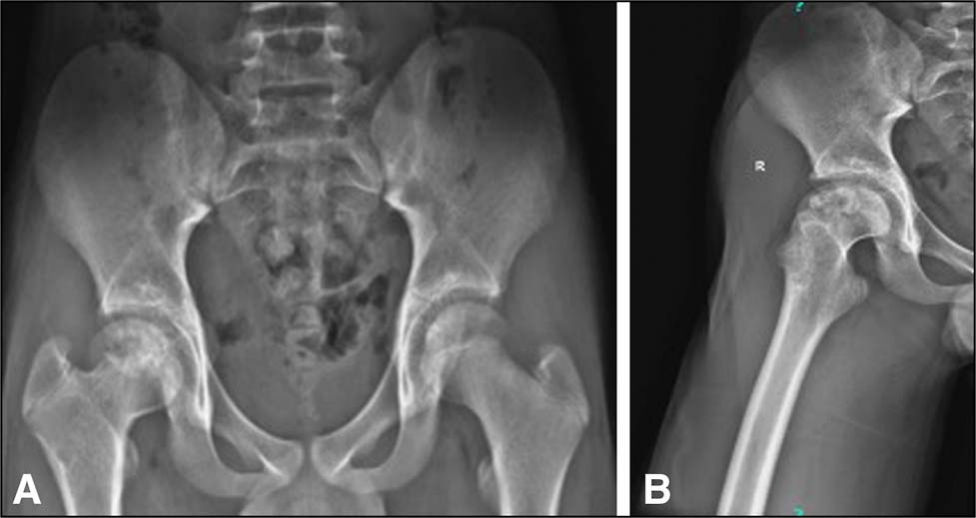
AP radiographs of the (**A**) pelvis and (**B**) lateral right hip 5 years after surgery show a concentric femoral head without evidence of recurrence and early closure of the epiphysis.

The patient was restricted to toe-touch weight-bearing for 2 months, when he was advanced to full weight-bearing. At his 3-year follow-up appointment, he reported no pain; ambulated with a non-antalgic gait; and had full, painless active and passive range of motion of the hip. Radiographically, he has no evidence of recurrence of the chondroblastoma, and he maintains a concentrically reduced femoral head without osteonecrosis. He does, however, have evidence of early physeal closure and a leg-length discrepancy of 1.1 cm (Figure 7). Five years after surgery, at age 15 years, he has returned to all activities of daily life; is asymptomatic with full, unrestricted, painless range of motion of the hip; and continues to play competitive hockey.

## Discussion

Femoral head lesions can be difficult to approach surgically. In some cases, surgical hip dislocation is necessary, using curettage and grafting through a transfoveal approach.^7^ Surgical hip dislocation in an adolescent can cause complications, such as premature arthritis, ischemic changes to the femoral neck, epiphyseal necrosis, premature epiphyseal plate arrest, and limb-length discrepancy.^8,9^ One study reported rates of osteonecrosis ranging from 0% to 26% in adults who underwent a modified Dunn procedure to treat slipped capital femoral epiphysis,^10^ but this complication has not been studied widely in children. However, in a study of medial open reduction in children with developmental dysplasia of the hip, 24% developed clinically relevant osteonecrosis at long-term follow-up.^11^

The capital epiphysis fuses at approximately 14 years in girls and 15 years in boys.^9^ Patients younger than these ages are at higher risk of premature epiphyseal plate arrest. In a study of 14 patients aged 7 to 32 years who underwent chondroblastoma removal using curettage and cryosurgery, the only two cases of epiphyseal plate arrest occurred in the two youngest patients, a 7-year-old boy and 10-year-old girl.^12^ Thus, precautions must be taken in young patients to minimize epiphyseal plate arrest. The use of arthroscopic techniques for the treatment of chondroblastoma has been reported in a patient with a lesion in the distal femur.^13^ We have avoided such arthroscopic approaches because of the potential for intra-articular spreading of tumor and resultant widespread soft-tissue recurrence due to spillage of tumor cells during surgery.^14^ In this case, while an arthroscopic bone tunnel would enable visualization of the cavity, it would not permit removal of the tumor. Arthroscopy through a bone tunnel could be considered to assess the completeness of the tumor removal.

Radiofrequency ablation has been used to treat chondroblastoma. However, this technique is effective in small lesions only and has been associated with major risk of osteonecrosis, collapse of the femoral head, and local recurrence.^15^

Despite an extensive review of the English-language literature, we were unable to find reports using the X-REAM in this manner. The only use described is for débridement of necrotic bone in the femoral head in patients with osteonecrosis. The X-REAM percutaneous expandable reamer, an advanced decompression tool, can be used to excise chondroblastomas. After insertion into the site of interest, the reamer is rotated until the desired expansion of the blade is obtained. The reamer's position is observed under fluoroscopic imaging in lateral and AP views. After the blade is removed, a suction tip is inserted to irrigate the system and remove the tumor. An injectable graft can be placed to fill the core on completion of tumor removal. The X-REAM was designed to “efficiently facilitate standard core decompression,”^16^ providing a less invasive technique that still produces sufficient tissue removal. Although the technique described avoids the complications associated with dislocation of the hip, our patient experienced early physeal closure with resultant limb-length discrepancy and obvious radiographic changes of the femoral head. Given his age, he is unlikely to need treatment of the limb-length discrepancy. However, had he been younger, it is likely that the limb-length discrepancy would have needed treatment. Finally, although local tumor control was obtained in this case, we know that local recurrence depends not only on the adequacy of curettage but also on the biological characteristics of the tumor. Because there are no predictors of biological behavior, it seems reasonable to treat selected cases of chondroblastoma of the proximal femoral epiphysis with the X-REAM as described in this study in younger patients to avoid surgical hip dislocation while accepting the consequence of early epiphyseal plate closure. This treatment may enable the surgeon to “buy time” and perform a more direct and open procedure if there is a recurrence when the patient is older and has less risk of complications associated with hip dislocation.

## Conclusion

This case describes a unique approach to the problems presented by the epiphyseal location of chondroblastoma. Growth arrest and limb-length discrepancy are risks of any surgical procedure that violates the physis. The technique described enables extended curettage of the epiphyseal lesion with minimal insult to the physis. It also obviates the need for surgical hip dislocation and trap-door excision of the lesion, which increases the risk of osteonecrosis.^10^ Incomplete excision leading to recurrence of chondroblastoma is a common pitfall when treating such lesions. Five years postoperatively, the patient has experienced no recurrence of his lesion, indicating that this minimally invasive approach achieved curettage while limiting physeal and soft-tissue trauma. For patients with chondroblastoma of the proximal femoral epiphysis, local control can be achieved without surgical dislocation of the hip or thermal ablation by using a cannulated expandable reamer.
